# Associations amongst dynamic knee stiffness during gait, quadriceps stiffness, and the incidence of knee osteoarthritis over 24 months: a cohort study with a mediation analysis

**DOI:** 10.1186/s12891-024-07618-4

**Published:** 2024-07-03

**Authors:** Zongpan Li, Kam-Lun Leung, Chen Huang, Xiuping Huang, Shan Su, Raymond CK Chung, Changhai Ding, Siu-Ngor Fu

**Affiliations:** 1 Department of Rehabilitation Sciences, The Hong Kong Polytechnic University, 11 Yuk Choi Road, Hong Kong, Hong Kong China; 2 Clinical Research Centre, Zhujiang Hospital, Southern Medical University, Guangzhou, Guangdong China

**Keywords:** Dynamic knee stiffness, Quadriceps stiffness, Gait, Knee osteoarthritis

## Abstract

**Background:**

Decreased strength and increased stiffness of the quadriceps have been associated with a higher risk of developing knee osteoarthritis (OA) in elders. Dynamic joint stiffness (DJS) represents collective resistance from active and passive knee structures for dynamic knee motions. Elevated sagittal knee DJS has been associated with worsening of cartilage loss in knee OA patients. Altered quadriceps properties may affect DJS, which could be a mediator for associations between quadriceps properties and knee OA. Hence, this study aimed to examine whether DJS and quadriceps properties would be associated with the development of clinical knee OA over 24 months, and to explore the mediation role of DJS in associations between quadriceps properties and knee OA.

**Methods:**

This was a prospective cohort study with 162 healthy community-dwelling elders. Gait analysis was conducted to compute DJS during the loading response phase. Quadriceps strength and stiffness were evaluated using a Cybex dynamometer and shear-wave ultrasound elastography, respectively. Knee OA was defined based on clinical criteria 24 months later. Logistic regression with generalized estimating equations was used to examine the association between quadriceps properties and DJS and incident knee OA. Mediation analysis was performed to explore the mediation role of DJS in associations between quadriceps properties and the incidence of knee OA.

**Results:**

A total of 125 participants (65.6 ± 4.0 years, 58.4% females) completed the 24-month follow-up, with 36 out of 250 knees identified as clinical knee OA. Higher DJS (OR = 1.86, 95%CI: 1.33–2.62), lower quadriceps strength (1.85, 1.05–3.23), and greater quadriceps stiffness (1.56, 1.10–2.21) were significantly associated with a higher risk of clinical knee OA. Mediation analysis showed that the DJS was not a significant mediator for the associations between quadriceps properties and knee OA.

**Conclusions:**

Higher sagittal knee dynamic joint stiffness, lower quadriceps strength, and greater quadriceps stiffness are potential risk factors for developing clinical knee OA in asymptomatic elders. Associations between quadriceps properties and knee OA may not be mediated by dynamic joint stiffness. Interventions for reducing increased passive properties of the quadriceps and knee joint stiffness may be beneficial for maintaining healthy knees in the aging population.

**Supplementary Information:**

The online version contains supplementary material available at 10.1186/s12891-024-07618-4.

## Background

Knee osteoarthritis (OA) develops slowly along a continuum from early joint vulnerability to established OA [1]. Mechanics are important factors related to the development of knee OA [2]. Quadriceps weakness has been one of the well-studied mechanical factors, related to the onset of knee OA [3]. The underpinning mechanism is thought to be that weak quadriceps may generate insufficient internal force to protect the knee joint from mechanical damages [4].

As optimal muscle function depends on both good active and passive properties, increased muscle passive stiffness may hamper optimal muscle function [5, 6]. Indeed, higher passive stiffness of the quadriceps has been found to be associated with a higher risk of developing clinical knee OA in 12 months in asymptomatic older adults [7]. The loading response phase of human gait, immediately following the initial contact of the heel with the ground, induces an abrupt and large loading impact on the lower limb joints [8]. During this critical phase, the quadriceps muscle functions eccentrically to control knee flexion excursion, attenuating the impact of knee loading [8, 9]. Inter-segmental movement is achieved by contractions of skeletal muscles and deformations of the peri-articular structures, which generate resistance to joint movements and produce passive joint moments [10]. The quadriceps muscle spans the knee joint via the muscle–tendon complex system. An increase in passive stiffness of the quadriceps, characterised by reduced compliance [11], may contribute to a high level of sagittal knee dynamic knee stiffness (DJS) [12, 13]. It reflects the collective biomechanical effects of active and passive knee structures on dynamic knee angular-joint stiffness and is identified as knee joint “quasi-stiffness” [12, 14], representing the collective resistance applied onto the knee joint during dynamic motions [15]. Whether quadriceps strength and stiffness would be associated with DJS have not been established. This would enhance our understanding of the potential mechanism by which altered quadriceps properties may contribute to the development of knee OA via change in joint stiffness.

Patients with established knee OA demonstrated elevated sagittal knee DJS than healthy controls [15, 16]. In addition, a prospective study from Chang et al. (2017) found that higher sagittal knee DJS during the loading response phase of gait was associated with the worsening of cartilage damage over a period of 2 years in knee OA patients [13]. Greater sagittal knee DJS may restrict sagittal knee motion concentrating load to a smaller knee joint surface [14, 15], thus may contribute to the development of knee OA. Hence, it is important to investigate whether DJS would predict the incidence of knee OA, and its potential mediation role in the relationship between quadriceps properties and knee OA onset.

The aims of this study were: (i) to examine the association between sagittal knee DJS with the risk of developing clinical knee OA over 24 months; (ii) to delineate the relationship between quadriceps strength/stiffness and sagittal knee DJS; (iii) to explore the mediation role of sagittal knee DJS in the association between quadriceps strength/stiffness and the incidence of clinical knee OA. We hypothesized that: (i) higher sagittal knee DJS would be associated with a higher risk of developing clinical knee OA over 24 months; (ii) lower strength and higher stiffness of the quadriceps would be correlated with higher sagittal knee DJS; (iii) the association between quadriceps properties and the risk of knee OA would be mediated by the sagittal knee DJS.

## Methods

### Study design and sample

This was a prospective cohort study. This cohort has been described in the previous publications [7, 17]. Participants were recruited from local communities in Hong Kong between November 2018 to October 2019. Potential participants were included if they were (i) aged 60–80 years, and (ii) able to walk 10 m without using an assistive device. Participants were excluded if they had (i) knee pain or known arthritis, (ii) history of knee injury or surgery, (iii) knee or hip joint replacement, (iv) cognitive impairment, or (v) a neurological condition (e.g., stroke or Parkinson’s disease). This study complies with the Declaration of Helsinki, that the locally appointed ethics committee has approved the research protocol and that written informed consent has been obtained from the participants (ID no.: HSEARS20180110001).

### Demographics

At baseline, age, sex, body mass, body height, and body mass index (BMI) were collected from all eligible participants. Known comorbidities (hypertension, diabetes, cardiopulmonary disease, and dyslipidaemia) were also documented. Each participant was categorized as having a sedentary or active lifestyle based on the type and duration of physical exercises they undertook, as recommended by the U.S. Department of Health and Human Services [18]. The Montreal Cognitive Assessment was used to screen for the presence of mild cognitive impairment, based on a cut-off score of 22 [19].

### Quadriceps strength at baseline

The isokinetic peak torque of the quadriceps (for both limbs) was assessed at a constant angular velocity of 60°/s using a Cybex isokinetic dynamometer (Cybex Co., Ronkonkoma, NY, USA) [7, 20]. Participants were placed in a sitting position and instructed to concentrically move their knee from maximum knee flexion to full extension with maximal effort, and to repeat this movement five times. A warm-up trial with 50% maximal voluntary effort was provided and verbal encouragement was given during the tests. The peak torque values of the middle three trials of knee extension (quadriceps strength) were averaged and normalized to body mass (Nm/kg) [7, 20]. Test-retest reliability was 0.90 for 20 healthy older adults (50% females) with a similar age and BMI [7].

### Quadriceps stiffness at baseline

Shear-wave ultrasound elastography (SWUE) quantifies the propagation speed of shear waves in skeletal muscles [21] and it is a valid and reliable method to estimate the stiffness of skeletal muscles [22–24]. Muscle shear moduli, an index of muscle stiffness [22–24], of the three superficial muscle heads (rectus femoris [RF], vastus lateralis [VL], and vastus medialis [VM]) (for both limbs) were assessed using a SWUE system equipped with an Aixplorer ultrasound scanner (Aixplorer Version 4.2; Supersonic Imagine, Aix-en-Provence, France), coupled with a linear ultrasound probe (4–15 MHz, Super Liner 15 − 4; Supersonic Imagine), following a previously established protocol [7, 25].

The room temperature was controlled at 25 °C during the measurements. Participants were in a supine position with their knees at 60° flexion and their hips in a neutral position with the aid of an adjustable brace. This measurement position was chosen to ensure that muscle fibers were beyond the slack position [25]. The measurement sites were located halfway between the anterior superior iliac spine (ASIS) and the patella superior border for the RF; distally one-third between the ASIS and the patella lateral border for the VL; and distally one-fifth between the ASIS and the patella medial border for the VM [7, 25].

Ultrasound gel was applied to the skin, and minimal ultrasound-probe pressure was used to avoid compressing the underlying muscle. An 11-s video (1 sample/s with a spatial resolution of 1 × 1 mm) was captured when the tested muscle was resting (as determined by inspection of the ultrasound images). Each video was transformed into 11 frames, with the middle five frames averaged for analysis. The region of interest (a two-dimensional area of the muscle shear modulus) was delineated, based on the muscle thickness underneath the marked site. The shear modulus values (in kPa) from each muscle head were computed using MATLAB (MathWorks, Inc, Natick, MA, USA). We summed the shear modulus of RF, VL, and VM to reflect the overall stiffness of the total superficial quadriceps [7, 26]. All the measurements were conducted by a single examiner (LZP). The intra-rater reliabilities were 0.94, 0.89, and 0.94 for the RF, VL, and VM, respectively, for 20 healthy older adults (50% females) with a similar age and BMI [7].

### Gait analysis at baseline

The gait analysis was conducted at a motion and gait laboratory. An eight-camera (MX T40) Motion Analysis System (Vicon, Oxford, UK) with two floor-mounted force plates (Advanced Mechanical Technology Inc., Watertown, MA, USA) were used to capture three-dimensional motion and knee moments. Sampling rates of 1,000 and 100 Hz were used to capture the kinetic and kinematic data, respectively. The standard Plug-In-Gait marker set was applied to the pelvic, hip, knee, and ankle joints of both lower limbs [16, 17]. Each participant walked unshod and without any assistive device on a 10-m walkway at a self-selected comfortable speed. During three practice trials, we instructed participants to find their self-selected comfortable walking speed and to maintain the same speed during the actual tests. During the testing, if the walking speed was obviously deviated from the self-selected speed, the participant was given a reminder, and this trial was not used for analysis. A successful trial was defined as clean foot strikes on the force plates of each leg. Five successful trials were captured and saved for off-line analyses.

**Fig. 1:**
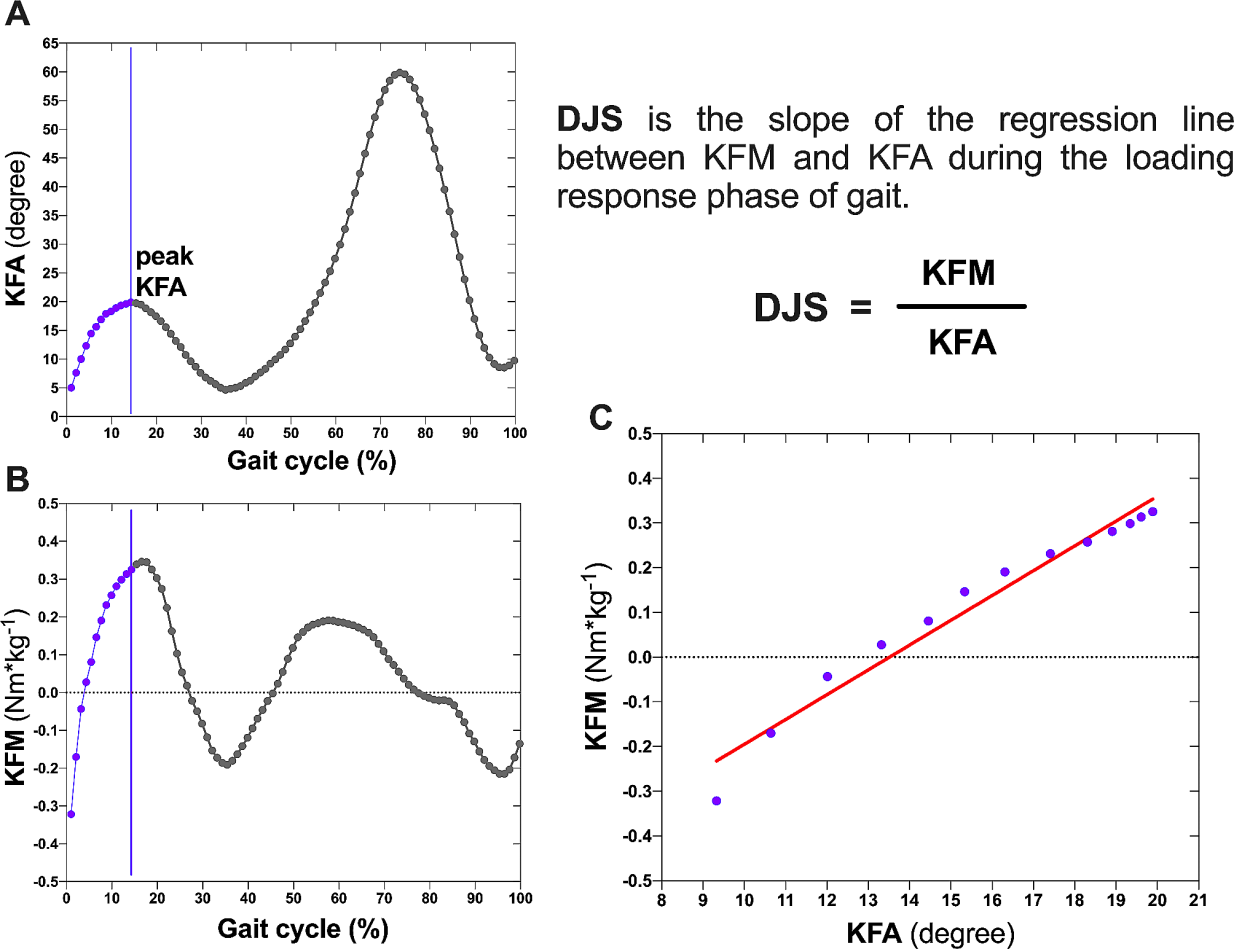
The calculation of sagittal knee DJS. DJS: dynamic joint stiffness; KFA: knee flexion angle; KFM: knee flexion moment

Nexus software (Version 2.5, Vicon) was used to analyze the knee joint kinetic and kinematic data for both limbs. A vertical ground reaction force threshold of 20 N was used to identify heel contact and toe-off events [27]. A custom MATLAB program (MathWorks, Inc, Natick, MA, USA) was used to compute knee biomechanical outcomes. The loading response phase was identified as the time from initial contact to the early stance peak knee flexion angle, during which the moment-angle curve is approximately linear (Fig. 1) [13, 15, 16]. The sagittal knee DJS (Nm·kg^− 1^/degree) was calculated as the slope of the linear regression line of the change in knee flexion moment against the change in knee flexion angle during the loading response. Walking speed was also recorded for all participants. The DJS was normalized to body mass [16, 17]. Each parameter was averaged over five successful trials for analysis.

### Incidence of clinical knee OA at 24 months

All the participants were invited for follow-up visits 24 months after their first consultation. Clinical examinations were conducted by a physiotherapist (HXP) with 8 years of post-qualification experience. Clinical examinations of the knee joint (its bony enlargement, warmth, bony tenderness, and crepitus) were performed based on a standardized protocol [7, 28]. Clinical knee OA was defined based on the clinical criteria from American College of Rheumatology [29]. As all the participants were aged over 50 years, they were regarded as having clinical knee OA if they had pain in the knee and any two of the following items: (i) morning stiffness for less than 30 min; (ii) crepitus; (iii) bony tenderness; (iv) bony enlargement; or (v) no palpable warmth of the synovium. The pain intensity (numeric rating scale, NRS), related activities causing knee pain, and the region of knee pain were also documented. The assessor and all the participants were blinded to the baseline measurements.

### Statistical analysis

Logistic regression was used to examine the associations between baseline sagittal knee DJS and quadriceps properties (quadriceps strength, passive stiffness of the total superficial quadriceps, RF, VL, and VM) (continuous outcomes) and the development of clinical knee OA over 24 months (dichotomous outcome) with and without the adjustment of age, sex, BMI (for quadriceps stiffness only) or body height (for quadriceps strength and DJS), comorbidities, activity level, and walking speed (for DJS). The body height would be entered into the adjusted models with quadriceps strength or DJS, as they had been normalized to body mass [7, 16, 17, 20]. In addition, quadriceps properties and DJS were further entered into the multivariable models, to assess their associations with clinical knee OA with the adjustments by each other. We used generalized estimating equations to control for between-knee correlations within each participant. For each logistic regression model, the odds ratio (OR), 95% confidence interval (95% CI), and corresponding *P* value were reported.

Sensitivity analyses were conducted by using the person-level data (*n* = 125 participants). For each participant, the DJS and quadriceps properties were averaged from both limbs. The incidence of clinical knee OA was determined as clinical knee OA on either knee. Logistic regression was conducted to examine the associations of baseline sagittal knee DJS and quadriceps properties with the incidence of clinical knee OA over 24 months, with and without controlling for the covariates as mentioned above.

Associations between quadriceps properties (strength and stiffness) and sagittal knee DJS were examined by linear regression, with and without controlling for age, sex, body height, comorbidities, activity level, and walking speed. For each linear regression model, the coefficients (β), 95%CI, coefficient of determination (R^2^), and related *P* value were computed. The R^2^ was interpreted based on the recommendations from J. Cohen (1988): Very weak: R^2^ < 0.02; Weak: 0.02 ≤ R^2^ < 0.13; Moderate: 0.13 ≤ R^2^ < 0.26; Substantial: R^2^ ≥ 0.26 [30].

**Fig. 2:**
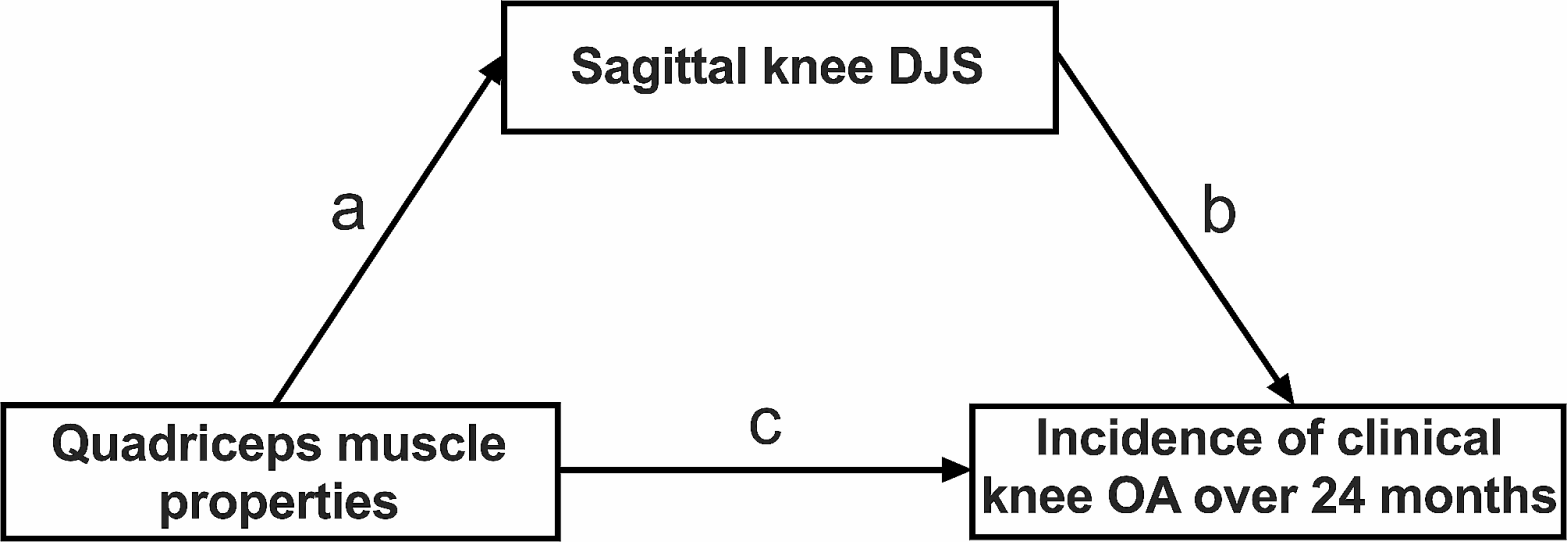
Conceptualization of mediation analyses. DJS: dynamic joint stiffness; OA: osteoarthritis

Mediation analysis was performed to examine the possible mediation role of sagittal knee DJS in the association between quadriceps properties (strength and stiffness) and the incidence of clinical knee OA over 24 months, controlling for the covariates as mentioned above (Fig. 2). The analyses were conducted using R statistical software (Version 4.1.2). A structural equation modeling program accomplished with Diagonally Weighted Least Squares were used to handle the dichotomous outcome (Lavaan 0.6-9) [31, 32]. The direct (DE: c) and indirect (IE: a*b) effects with 95% CI and corresponding *P* values were computed for each model. Partial mediation was defined if indirect and direct effects were all significant, while complete mediation was considered as a significant indirect effect but a nonsignificant direct effect [33].

All statistical significances were pre-set at *P* < 0.05 (two-tailed).

## Results

### Cohort descriptions

**Fig. 3:**
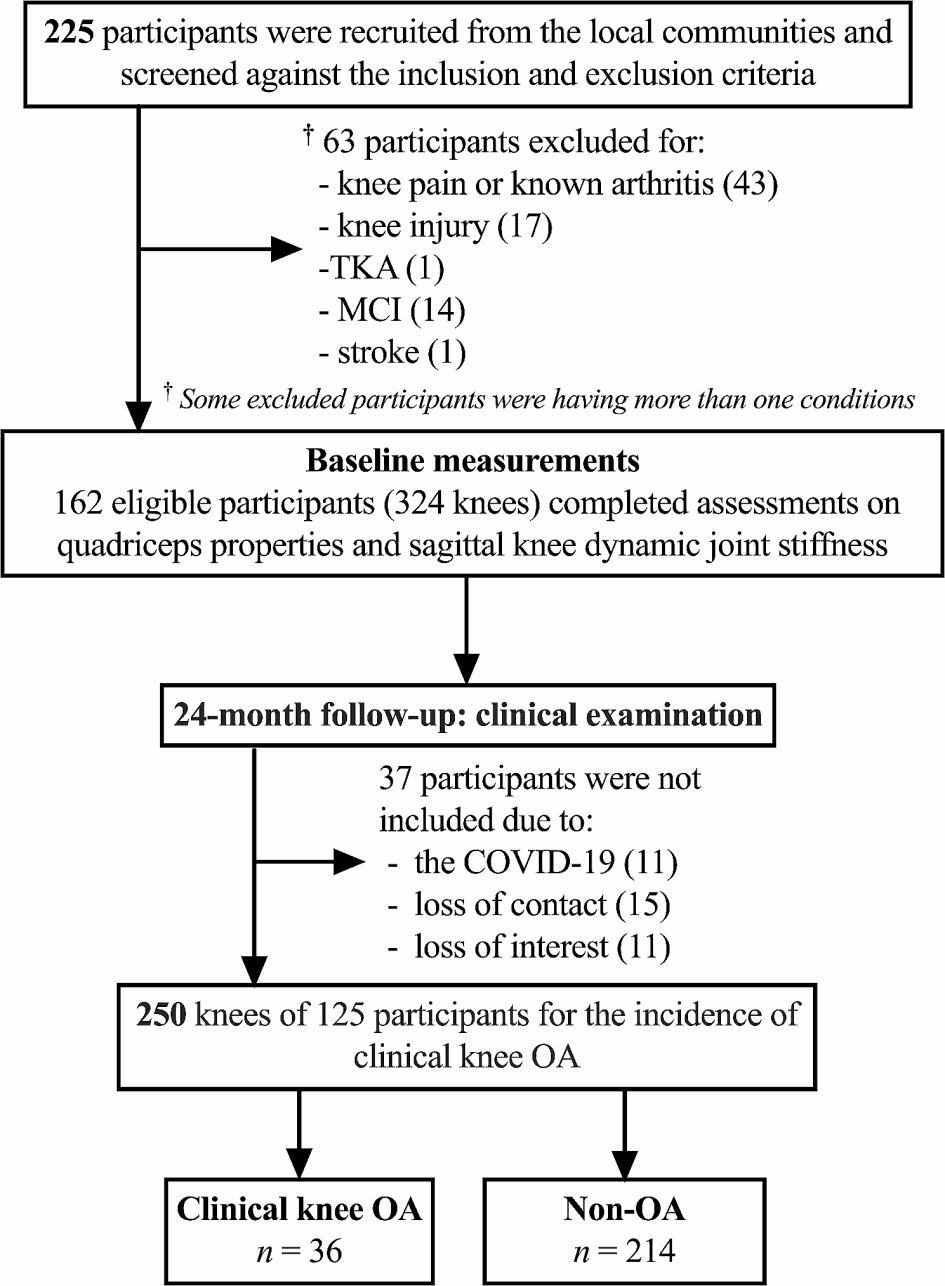
Participant inclusion diagram. TKA: total knee arthroplasty; MCI: mild cognitive impairment; OA: osteoarthritis

Two hundred and twenty-five potential participants were screened, and 63 of them were excluded because of knee pain, known arthritis, knee injury, joint replacement, mild cognitive impairment, or stroke. Baseline assessments were conducted from November 2018 to October 2019, and all outcome measures were available for the 162 eligible participants (61.1% females, age: 65.8 ± 4.0 years). The participant inclusion diagram is shown in Fig. 3. One hundred and twenty-five participants were included in the analysis of clinical knee OA at 24 months, with 37 others were dropped due to the COVID-19, loss of contact, or loss of interest. The demographics and descriptive data from baseline measurements are presented in Table 1. No significant differences in demographic data, muscle stiffness, or gait measurements were found between those who were included in the analyses and those who dropped out at 24 months (*P* = 0.193–0.968).

**Table 1:** Baseline characteristics

	162 participants tested at baseline (*n* = 324 knees)	125 participants included at 24 months (*n* = 250 knees)	37 participants excluded at 24 months (*n* = 74 knees)
Demographics			
Age (years)	65.8 ± 4.0	65.6 ± 4.0	66.5 ± 4.0
Sex (Females: Males)	99: 63	73: 52	26: 11
Body mass (kg)	58.7 ± 10.0	59.2 ± 9.7	57.1 ± 11.1
Body height (m)	1.59 ± 0.08	1.60 ± 0.08	1.56 ± 0.08
Body mass index (kg/m^2^)	23.2 ± 3.4	23.2 ± 3.2	23.3 ± 3.9
Activity level (Sedentary: Active)	83: 79	63: 62	20: 17
^†^***n *of comorbidities**: *n* (%) of participants	**0**: 92 (56.8%) **1**: 44 (27.2%) **>1**: 26 (16.0%)	**0**: 73 (58.4%) **1**: 32 (25.6%) **>1**: 20 (16.0%)	**0**: 19 (51.4%) **1**: 12 (32.4%) **> 1**: 6 (16.2%)
Walking speed (m/s)	1.19 ± 0.18	1.19 ± 0.17	1.19 ± 0.20
DJS (Nm*kg ^**− 1**^ **/degree)** ^‡^	0.066 ± 0.036	0.066 ± 0.036	0.066 ± 0.036
Change in knee flexion moment (Nm*kg^− 1^)	0.499 ± 0.300	0.491 ± 0.295	0.526 ± 0.318
Change in knee flexion angle (degree)	8.391 ± 3.730	8.464 ± 3.825	8.147 ± 3.405
**Quadriceps properties** ^‡^			
Quadriceps strength (Nm/kg)	1.27 ± 0.36	1.29 ± 0.36	1.23 ± 0.35
Total quadriceps stiffness (kPa)	21.4 ± 4.8	21.3 ± 4.9	21.7 ± 4.5
Rectus femoris stiffness (kPa)	11.2 ± 3.4	11.1 ± 3.4	11.4 ± 3.2
Vastus lateralis stiffness (kPa)	5.6 ± 1.6	5.6 ± 1.5	5.8 ± 1.6
Vastus medialis stiffness (kPa)	4.6 ± 0.9	4.6 ± 0.9	4.6 ± 0.9

Values are mean ± SD or numbers (*n*) unless other indicates; DJS: sagittal knee dynamic joint stiffness during loading response phase; ^†^ including hypertension, diabetes, cardiopulmonary disease, and dyslipidaemia. ^‡^ DJS and quadriceps properties included both knees of each participant

### Incidence of clinical knee OA over 24 months

The 24-month incidence of clinical knee OA was 19.2% (*n* = 24/125 participants), with 11 participants developed bilateral clinical knee OA. The knee-level analyses included 250 knees, among which 36 knees were defined as clinical knee OA. The overall pain intensity for the OA knees was 5.2 ± 1.3 on NRS. The painful regions were evenly distributed at anterior (47.2%), medial (44.4%), and lateral (44.4%) knee, followed by posterior knee (22.2%). The painful region for over half of the cases was restricted at one site (58.3%), with other 11 knees (30.6%) at 2 sites and 4 knees (11.1%) at multiple sites (≥ 3 sites). Knee pain was most often present during stair climbing (86.1%), walking up and down slopes (69.4%), and squatting (63.9%), followed by kneeling (52.8%), walking (44.4%), jumping (33.3%), and running (30.6%). Crepitus was the most common clinical sign (88.9%), followed by morning stiffness for less than 30 min (72.2%) and bony tenderness (25.0%). Bony enlargement was rare (2.8%), and no significant warmness was detected around the knee synovium of any participants.

### DJS and clinical knee OA

A higher sagittal knee DJS was significantly associated with a higher risk of developing clinical knee OA in 24 months prior to (OR = 1.51; 95% CI: 1.06–2.14; *P* = 0.022) and after (adjusted OR = 1.55; 95% CI: 1.10–2.17; *P* = 0.011) the adjustment of the covariates (Table 2). When further adjusted by quadriceps strength and stiffness, the significant association persisted (adjusted OR = 1.86; 95% CI: 1.33–2.62; *P* < 0.001).

**Table 2:** Associations of baseline DJS and quadriceps properties with clinical knee OA over 24 months

	Univariable				Multivariable ^†^				Multivariable ^‡^	
	OR [95% CI]	*P* value		OR [95% CI]		*P* value		OR [95% CI]		*P* value
**Sagittal knee DJS**	**1.51 [1.06–2.14]**	**0.022**		**1.55 [1.10–2.17]**		**0.011**		**1.86 [1.33–2.62]**		**< 0.001**
**Quadriceps properties**										
Quadriceps strength	**1.57 [1.01–2.44]**	**0.049**		1.62 [0.93–2.81]		0.086		**1.85 [1.05–3.23]**		**0.032**
Total quadriceps stiffness	**1.38 [1.02–1.87]**	**0.038**		**1.41 [1.02–1.93]**		**0.035**		**1.56 [1.10–2.21]**		**0.014**
Rectus femoris stiffness	1.34 [0.97–1.85]	0.078		1.41 [1.00–2.01]		0.052		**1.44 [1.02–2.05]**		**0.040**
Vastus lateralis stiffness	1.23 [0.83–1.83]	0.311		1.20 [0.82–1.75]		0.354		1.34 [0.88–2.04]		0.179
Vastus medialis stiffness	1.33 [0.94–1.88]	0.111		1.26 [0.89–1.80]		0.191		**1.53 [1.04–2.25]**		**0.031**

OA: osteoarthritis; DJS: dynamic joint stiffness; ^**†**^ adjusted by age, sex, body mass index (body height for quadriceps strength and DJS), comorbidities, activity level, and walking speed (for DJS); ^**‡**^ adjusted by age, sex, body height, comorbidities, activity level, walking speed, and quadriceps properties/DJS; OR: odds ratio; 95% CI: 95% confidence interval of OR; Significant association in Bold

Sensitivity analysis on the person-level data showed that higher DJS was significantly associated with a higher incidence of clinical knee OA before (OR = 2.10; 95% CI: 1.26–3.49; *P* = 0.004) and after (adjusted OR = 2.15; 95% CI: 1.24–3.74; *P* = 0.007) controlling for the covariates. Similarly, such significant association remained after further adjustment by quadriceps strength and stiffness (adjusted OR = 2.54; 95% CI: 1.42–4.56; *P* = 0.002) **[see Additional file 1]**.

### Quadriceps properties and clinical knee OA

Results of the logistic regression analyses indicated that lower quadriceps strength was significantly associated with a higher risk of developing clinical knee OA in 24 months (OR = 1.57; 95% CI: 1.01–2.44; *P* = 0.049). Higher stiffness of the total superficial quadriceps was significantly associated with a higher risk of developing clinical knee OA in 24 months before (OR = 1.38; 95% CI: 1.02–1.87; *P* = 0.038) and after (adjusted OR = 1.41; 95% CI: 1.02–1.93; *P* = 0.035) considering the covariates. After further adjusted by DJS, lower quadriceps strength (adjusted OR = 1.85; 95% CI: 1.05–3.23; *P* = 0.032), and higher stiffness of the total quadriceps (adjusted OR = 1.56; 95% CI: 1.10–2.21; *P* = 0.014), RF (adjusted OR = 1.44; 95% CI: 1.02–2.05; *P* = 0.040), and VM (adjusted OR = 1.53; 95% CI: 1.04–2.25; *P* = 0.031) were significantly associated with a higher risk for developing clinical knee OA over 24 months (Table 2).

### Quadriceps properties and DJS

No significant relationship was found between quadriceps strength and DJS **[see Additional file 2]**; or between quadriceps stiffness and DJS **[see Additional file 3]**.

### Mediation analysis

Results of mediation analyses are presented in Table 3. Significant direct effects on the incidence of clinical knee OA were observed for quadriceps strength (DE = − 0.309, *P* = 0.020), total quadriceps stiffness (DE = 0.303, *P* = 0.021) and RF stiffness (DE = 0.291, *P* = 0.022). However, no significant indirect path was observed before (*P* = 0.104–0.284) or after (*P* = 0.057–0.276) the adjustment by the covariates.

**Table 3:** Mediation by DJS on the associations between quadriceps properties and clinical knee OA.

Quadriceps properties	Effect	Unadjusted				^†^ Adjusted	
		Effect [95% CI]	*P* value		Effect [95% CI]		*P* value
Quadriceps strength	Direct	**-0.309 [-0.570, -0.049]**	**0.020**		**-0.430 [-0.772, -0.089]**		**0.014**
	Indirect	0.053 [-0.019, 0.126]	0.150		0.200 [-0.006, 0.407]		0.057
Total quadriceps stiffness	Direct	**0.303 [0.046, 0.560]**	**0.021**		**0.296 [0.011, 0.582]**		**0.042**
	Indirect	-0.072 [-0.159, 0.015]	0.104		-0.074 [-0.162, 0.014]		0.100
Rectus femoris stiffness	Direct	**0.291 [0.042, 0.540]**	**0.022**		0.278 [-0.008, 0.565]		0.057
	Indirect	-0.069 [-0.156, 0.018]	0.121		-0.070 [-0.156, 0.017]		0.114
Vastus lateralis stiffness	Direct	0.192 [-0.037, 0.421]	0.101		0.237 [-0.031, 0.506]		0.083
	Indirect	-0.041 [-0.113, 0.031]	0.265		-0.111 [-0.281, 0.059]		0.200
Vastus medialis stiffness	Direct	0.181 [-0.104, 0.466]	0.214		0.230 [-0.095, 0.555]		0.166
	Indirect	-0.046 [-0.130, 0.038]	0.284		-0.046 [-0.128, 0.037]		0.276

OA: osteoarthritis; DJS: dynamic joint stiffness; ^**†**^ adjusted by age, sex, body height, comorbidities, activity level, and walking speed; 95% CI: 95% confidence interval; Significant effect in Bold

## Discussion

Our study indicated that a higher sagittal knee DJS, lower quadriceps strength, and greater quadriceps stiffness at baseline were associated with higher risk of developing clinical knee OA over 24 months in asymptomatic older adults. The indirect effects were not significant, suggesting the associations between quadriceps properties and the risk of developing clinical knee OA may not be mediated by the sagittal knee DJS.

We observed the 24-month incidence of clinical knee OA was 19.2%. The annual incidence was generally higher than the incidences reported for radiographic, symptomatic, and self-reported knee OA [34]. The radiographic criteria for knee OA are based on evidence of osteophyte formation or narrowing of the joint space (grade ≥ 2 in the Kellgren-Lawrence [K-L] classification system) [35], which may fail to identify early-stage knee OA (suggested as K-L grade 0 or 1, but with clinical signs and symptoms) [36]. MRI studies have identified pre-radiographic tissue lesions in knees with K-L grades below 2 [37], which are associated with activity-related pain [38]. In the present study, the ACR clinical criteria were applied [29]. Most of the knees were painful during walking up or down stairs (86.1%), had crepitus (88.9%), and had morning stiffness for less than 30 min (72.2%), while clinical signs related to structural damage were uncommon (i.e., bony enlargement: 2.8%). It was suggested that the onset of self-reported knee pain during stair climbing is an important criterion for the clinical definition of early knee OA [39]. In the current study, knee pain was approximately evenly distributed between the anterior (47.2%), lateral (44.4%), and medial (44.4%) regions. Around half of the participants had knee pain located at one knee region (58.3%) but diffuse pain (i.e., ≥ 3 regions with knee pain [40]) was not common (11.1%). These results are different from those of a previous report on individuals with radiographically established knee OA (K-L grade ≥ 2), for whom the most frequent pain zone was around the medial joint line (75%) and diffuse knee pain was the most frequent symptom pattern [40]. As such, most of the OA knees identified over the 24-month follow-ups were likely in the early stage of disease.

The results of this study supported our hypothesis that elevated DJS would be associated with a greater risk of developing clinical knee OA over a 24-month period. In a previous study, DJS has been suggested to be a risk factor for the 24-month exacerbation of knee cartilage damage in people with established knee OA [13]. DJS reflects the biomechanical interplay between the knee flexion moment and the knee flexion angle during the loading response phase of gait [13, 15, 16]. An elevated DJS reflects increased collective resistance from muscles and soft tissues for dynamic knee motions (a “quasi-stiffer” knee joint) during dynamic motions [15]. Greater sagittal knee DJS could potentially limit the range of motion in the knee, thereby concentrating stress on a reduced surface area of the joint [14, 15], potentially contributing to the development of knee OA.

Our findings on the quadriceps properties are consistent with the previous reports on the associations of lower quadriceps strength [3] and higher quadriceps stiffness [7] with the higher risk of developing knee OA. It suggests that the reduced strength and increased stiffness of the quadriceps may both be detrimental factors related to the onset of knee OA in the aging population. Noting that along with the reduced muscle strength, quadriceps stiffness is increased with aging [5]. In addition to muscle strength, the altered passive property of quadriceps may be another aspect needing to be considered in the management of knee OA in the aging population. Our results showed that the relationships between quadriceps stiffness and clinical knee OA were specific to RF and VM, but not the VL. This could be attributed to the distinctive roles of individual quadriceps heads in joint mechanics. Yamagata et al. (2022) demonstrated that amongst the Vasti, the bi-articular RF has most significant influence on knee contact force during walking [41]. Another study found that the VL is primarily activated during pre-loading phase of gait [42]. Thus, we speculated that altered VL properties might be less likely to have significant mechanical impacts on the knee joint especially during the loading phase. That could be a reason why we did not observe a significant relationship between VL stiffness and clinical knee OA. However, the effect of quadriceps passive stiffness on knee loading may require further studies for investigation.

The surprising findings were that no significant relationship was observed between quadriceps properties and sagittal knee DJS. We originally hypothesized that a decrease in muscle strength and an increase in passive stiffness of the quadriceps may be correlated with increased knee joint stiffness via its muscle–tendon complex system or its connection with the ligamentous system around the knee joint [43, 44]. While the data did not support our hypothesis, it was speculated that the main determinations of DJS might be from other structures around the knee joint (e.g., tendon, ligament, joint capsule) [14] that requires future studies to further explore.

No significant indirect effect was observed regarding the mediation role of sagittal knee DJS in the associations between quadriceps properties and clinical knee OA. Hence, the quadriceps properties may contribute to the development of clinical knee OA through other pathways needing further study to explore. For example, recent studies suggest that altered qualities of skeletal muscles may be resulted from inflammatory processes that can cause an excessive buildup of extracellular matrix components [45, 46]. Nevertheless, the present study further confirmed that reduced strength and increased stiffness of the quadriceps were significant predictor for the incidence of clinical knee OA over 24 months. In addition to muscle strengthening, targeting on reducing quadriceps stiffness may also be beneficial for maintaining good knee health for the elderly. More importantly, we found that elevated sagittal knee DJS was a potential risk factor for the development of clinical knee OA in the aging population. Strategies for reducing sagittal knee DJS may be another direction for the prevention of knee OA in the aging population. Conceptually, the potential approaches could be targeted on decreasing tight surrounding tissues (e.g., tendon, ligament, joint capsule). However, dynamic knee stiffness is estimated from net knee joint moment that does not consider contributions from passive structures. Overall, the findings from the present project may suggest that, in addition to facilitating muscle strength, it may be important for the healthcare practitioners to consider reducing increased passive properties of the quadriceps and promoting knee joint mobilities for maintaining healthy knees in the aging population.

The present study has several limitations. Only the three superficial muscle heads were measured to represent quadriceps stiffness. The deep head, vastus intermedius muscle, was not measured because its deep location beneath the superficial heads results in poor reliability for shear-wave elastography measurements [25]. We previously acknowledged the limitations of planar radiography for identifying early OA and its discordance with clinical symptoms [47]. Hence, knee radiography was not performed at baseline or at the follow-up visits. Therefore, our findings may not be generalizable to structural damage to the knee or radiographic knee OA. Another potential limitation could be that we did not use a validated questionnaire to screen participants with knee pain related to inflammatory joint diseases. However, participants’ medical histories were reviewed to exclude those with known inflammatory joint diseases. This was important, as such inflammatory diseases may cause knee pain by other (non-OA) mechanisms. In addition, our follow-up surveys showed that no participant had newly diagnosed inflammatory diseases. Furthermore, the incidence of knee pain was defined as perceived pain during weight-bearing activities, which is typical OA-related knee symptoms [48]. In addition, we used net joint moments as surrogates of knee loading in the estimation of dynamic joint stiffness. Moreover, we only examined the predictor variables at baseline but not their change over 24 months, which may limit our interpretation of their role in knee OA incidence.

## Conclusions

Higher sagittal knee dynamic joint stiffness during loading response phase of gait, lower quadriceps strength, and higher quadriceps stiffness are associated with a higher risk of developing clinical knee OA in 24 months in asymptomatic elders. Associations between quadriceps properties and knee OA may not be mediated by sagittal knee dynamic joint stiffness. Interventions for reducing increased passive properties of the quadriceps and knee joint stiffness may be beneficial for maintaining healthy knees in the aging population.

### Electronic supplementary material

Below is the link to the electronic supplementary material.

Supplementary Material 1

## Data Availability

The data analyzed in the current study are available from the corresponding author on reasonable request.

## Abbreviations

ASIS: anterior superior iliac spine
β: coefficient
BMI: body mass index
CI: confidence interval
DE: direct effect
DJS: dynamic joint stiffness
IE: indirect effect
K-L: Kellgren-Lawrence
NRS: numeric rating scale
OA: Osteoarthritis
OR: odds ratio
RF: rectus femoris
R^2^: coefficient of determination
SWUE: shear-wave ultrasound elastography
VL: vastis lateralis
VM: vastis medialis
