# Supplementary material for: Associations amongst dynamic knee stiffness during gait, quadriceps stiffness, and the incidence of knee osteoarthritis over 24 months: a cohort study with a mediation analysis
Source: BMC Musculoskelet Disord. 2024 Jul 3;25:511. doi: 10.1186/s12891-024-07618-4 (PMC11220956; doi:10.1186/s12891-024-07618-4)
Supplement: Supplementary file 1 — Supplementary Material 1 [file 12891_2024_7618_MOESM1_ESM.docx]

**Additional material**

Additional file 1 Associations of baseline DJS and quadriceps properties with the incidence of clinical knee OA over 24 months.

|  | **Univariable** | |  | | **Multivariable ^†^** | |  | | **Multivariable ^‡^** | |
| --- | --- | --- | --- | --- | --- | --- | --- | --- | --- | --- |
|  | OR [95% CI] | *P* value | | OR [95% CI] | | *P* value | | OR [95% CI] | | *P* value |
| **Sagittal knee DJS** | **2.10 [1.26–3.49]** | **0.004** | | **2.15 [1.24–3.74]** | | **0.007** | | **2.54 [1.42–4.56]** | | **0.002** |
| **Quadriceps properties** | | | | | | | | | | |
| Quadriceps strength | 1.57 [0.97–2.56] | 0.067 | | 1.43 [0.79–2.57] | | 0.235 | | 1.73 [0.92–3.28] | | 0.091 |
| Total quadriceps stiffness | 1.48 [0.96–2.28] | 0.078 | | 1.45 [0.92–2.28] | | 0.108 | | **1.69 [1.03–2.80]** | | **0.040** |
| Rectus femoris stiffness | 1.46 [0.95–2.24] | 0.088 | | 1.50 [0.95–2.38] | | 0.085 | | **1.63 [1.00–2.65]** | | **0.048** |
| Vastus lateralis stiffness | 1.31 [0.86–2.02] | 0.214 | | 1.23 [0.79–1.94] | | 0.361 | | 1.38 [0.84–2.27] | | 0.197 |
| Vastus medialis stiffness | 1.25 [0.81–1.94] | 0.315 | | 1.18 [0.75–1.86] | | 0.463 | | 1.46 [0.89–2.40] | | 0.136 |
| OA: osteoarthritis; DJS: dynamic joint stiffness; **^†^** adjusted by age, sex, body mass index (body height for quadriceps strength and DJS), comorbidities, activity level, and walking speed (for DJS); **^‡^** adjusted by age, sex, body height, comorbidities, activity level, walking speed, and quadriceps properties/DJS; OR**:** odds ratio; 95% CI: 95% confidence interval of OR; Significant association in Bold. | | | | | | | | | | |

**
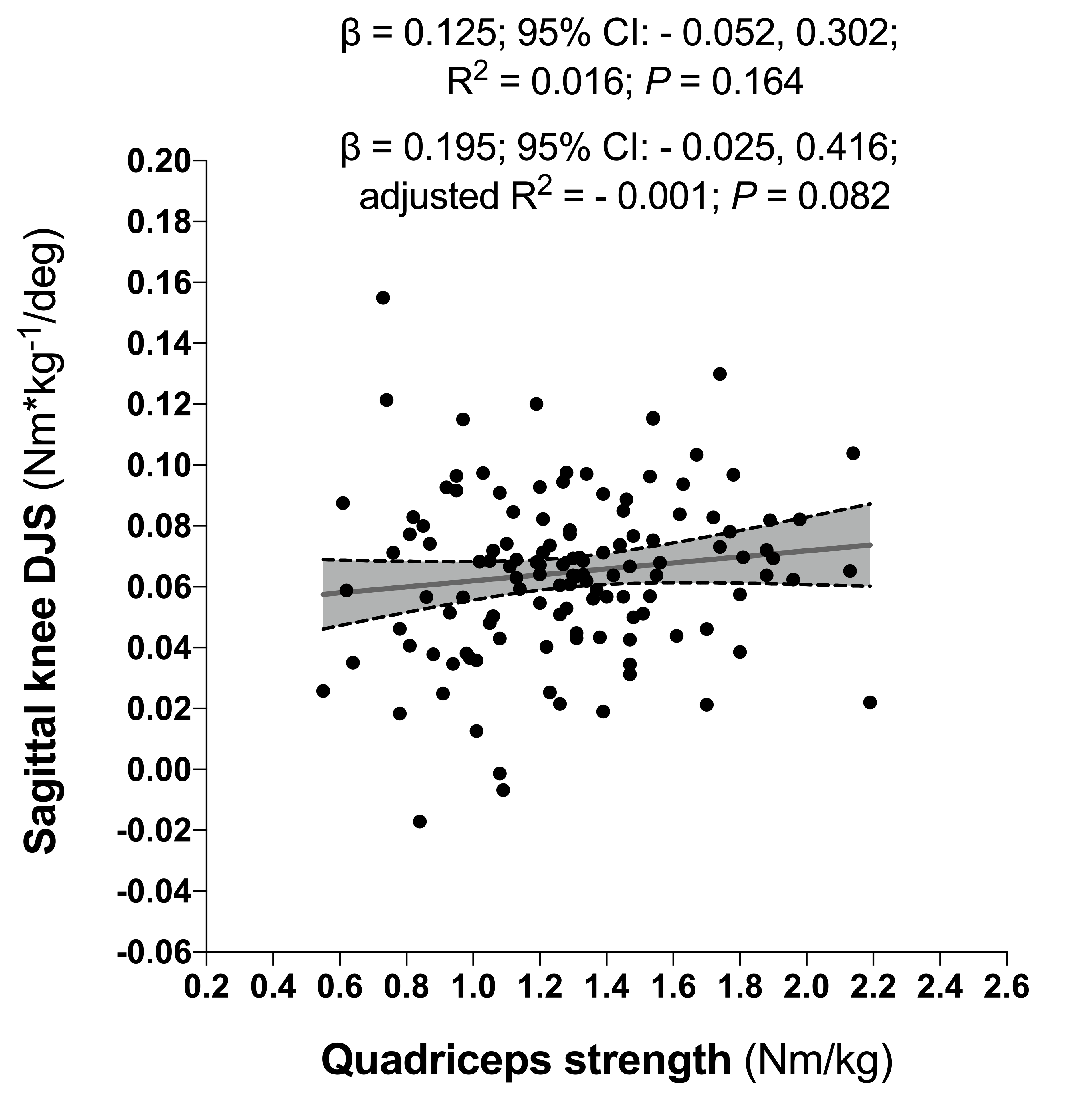
**

**Additional file 2** Relationships between quadriceps strength and sagittal knee DJS. DJS: dynamic joint stiffness; β: coefficients; 95% CI: 95% confidence interval.

**
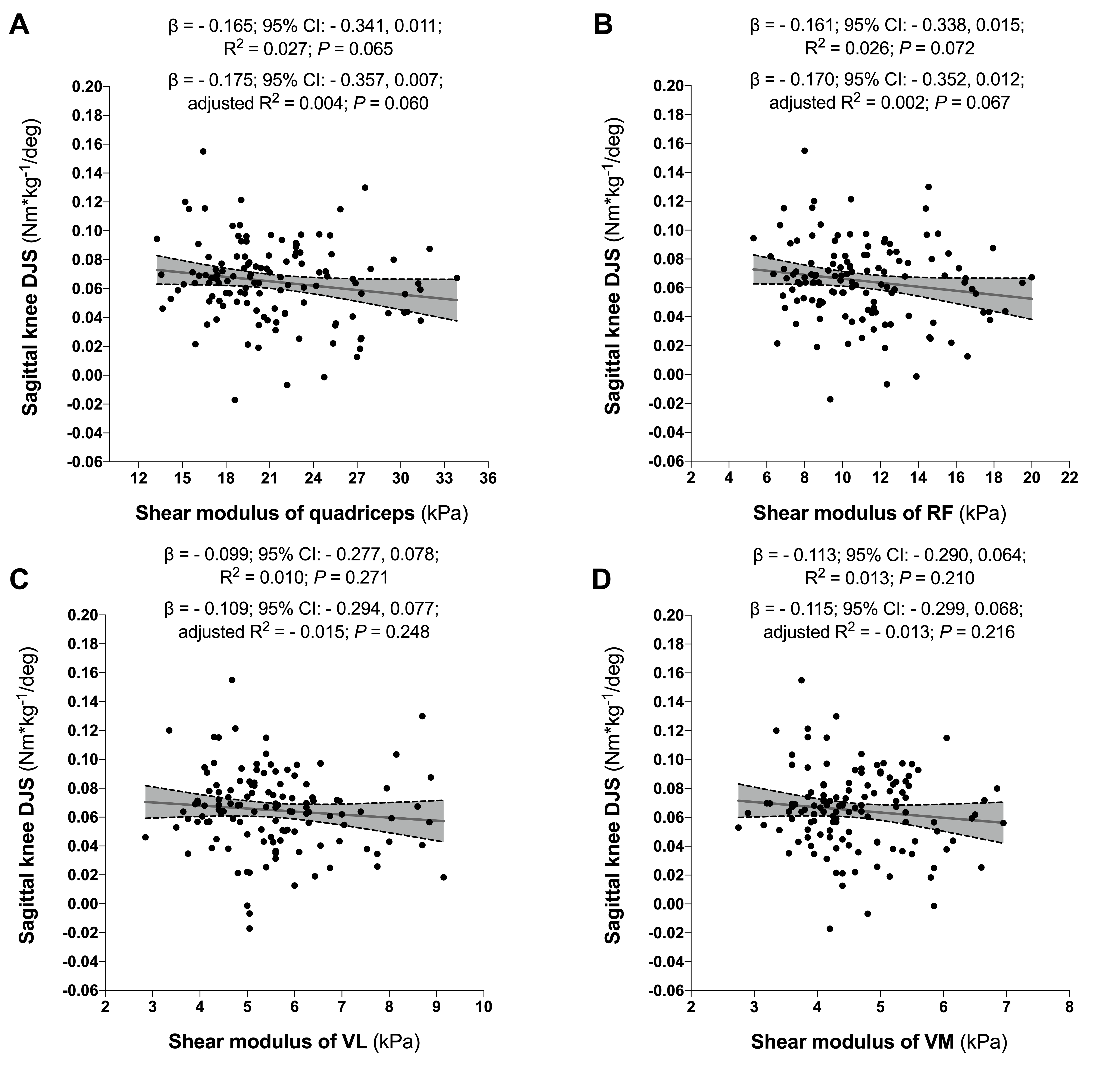
**

**Additional file 3** Relationships between quadriceps stiffness and sagittal knee DJS. DJS: dynamic joint stiffness; RF: rectus femoris; VL: vastus lateralis; VM: vastus medialis; β: coefficients; 95% CI: 95% confidence interval.
